# Potential of *Moringa oleifera* and *Moringa peregrina* whole plants as sustainable forage alternatives in lamb (*Ovis aries*) nutrition

**DOI:** 10.1038/s41598-025-31655-1

**Published:** 2025-12-07

**Authors:** Khalid A. Abdoun, Mohammed A. Al-Badwi, Faisal A. Alshamiry, Ahmed A. Alsagan, Mohammed Y. Alsaiady, Emad M. Samara, Ahmed A. Al-Haidary

**Affiliations:** 1 Department of Animal Production, College of Food and Agricultural Sciences, King Saud University, P.O. Box 2460, 11451 Riyadh, Saudi Arabia; 2 King Abdulaziz City for Science and Technology (KACST), 12354 Riyadh, Saudi Arabia

**Keywords:** Moringa, Sheep, Digestibility, Growth rate, Fermentation profile, Rumen discoloration, Biochemistry, Biological techniques, Biotechnology, Physiology, Plant sciences, Zoology

## Abstract

This study investigated the feasibility of replacing alfalfa hay with whole plants of *Moringa oleifera (MO)* and *Moringa peregrina (MP)* in pelleted total mixed rations (TMR) for growing Hari lambs under arid conditions. Sixty male lambs were allocated to five dietary treatments: T1) alfalfa-based control(, T2) MO-based(, T3 ) MP-based(, T4) alfalfa + MO( and T5) alfalfa + MP) over an 84-day feeding trial. Growth performance (ADG, final BW, and FCR) was not significantly affected (*P* > 0.05) by dietary inclusion of MO or MP, despite a significant reduction (*P* < 0.05) in average daily feed intake in MO treatments. Apparent nutrient digestibility of DM, CP, NDF, and ADF was unaffected (*P* > 0.05), except for a significant decrease (*P* < 0.05) in ether extract digestibility in Moringa diets. Rumen fermentation parameters revealed stable pH and total volatile fatty acid (VFA) concentrations across treatments; however, individual VFA profiles varied significantly (*P* < 0.05), with MP elevating acetic acid and branched-chain VFAs (isobutyrate, isovalerate), and MO increasing butyric acid concentrations. Colorimetric analysis of rumen epithelium demonstrated and association between increased lightness (L*) in Moringa-fed lambs and higher concentration of branched-chain VFAs (*r* = 0.551 and 0.487 for isobutyrate and isovalerate, respectively) and negatively correlated was observed with propionate (*r* = -0.793, *P* < 0.001). These data indicate that whole Moringa plants are effective alternative fibre sources capable of replacing alfalfa hay in pelleted lamb diets without detrimental effects on growth or major nutrient digestibility, while modulating ruminal fermentation pathways and epithelial tissue pigmentation. Further histopathological and biochemical investigations are required to elucidate the mechanisms underpinning rumen epithelial color changes associated with dietary Moringa inclusion.

## Introduction

Research in animal production has the potential to significantly boost GDP and enhance human health in developing countries. In Saudi Arabia, over 80% of irrigation water for crops like alfalfa (*Medicago sativa* L.) comes from limited and non-renewable groundwater sources. To address this issue, the Saudi government initiated a three-year program in 2016 aimed at conserving water resources by substantially reducing domestic crop production, including alfalfa, which is crucial for livestock. In this context, cultivating drought-tolerant crops is essential for maintaining agricultural sustainability. Moringa, a member of the Moringaceae family, thrives in tropical climates and is known for its drought tolerance^1^.

Nutritional analyses indicate that dried Moringa leaf powder offers protein and calcium levels comparable to powdered milk, and it is rich in vitamin A and powerful antioxidants^2^. Proximate analysis of *Moringa oleifera* leaves reveals higher crude protein and fat content, lower crude fibre, and elevated levels of calcium, iron, manganese, zinc, and selenium compared to alfalfa hay^3^. Moringa leaf meal has been found to provide similar protein and energy levels as other protein sources in dairy cows^4^. Replacing part of the alfalfa hay in dairy ewes’ diets with *Moringa oleifera* leaves has led to increased milk production^5^. Additionally, defatted Moringa leaves have improved fermentation and growth rates in lambs^6^. Moringa leaves show great potential as an alternative feed to soybean and rapeseed meal for ruminants^7^. Sanchez et al^8^. reported that incorporating Moringa as a protein supplement boosted milk production in Bosindicus. Moringa silage can be fed to dairy cows in large amounts to achieve similar milk yield and quality as traditional diets^4^. Although cows fed soybean meal produced more milk daily compared to those fed Moringa leaf meal or an optimized concentrate (13.2 kg vs. 12.3 kg and 12.1 kg, respectively), locally produced Moringa leaf meal can successfully replace commercial concentrate ingredients for dairy cows^4^.

As a protein alternative, 1.65 kg DM of Moringa leaf meal can replace 1.23 kg of cottonseed cake in dairy cows’ diets without impacting milk yield, while positively affecting the ruminal environment^9^. Including Moringa as a protein supplement in low-quality diets has been shown to improve dry matter intake and digestibility, thereby increasing milk production without altering milk composition^8^. Feeding *Moringa oleifera* leaves has also enhanced the productive performance of goat kids^10,11^, and growing lambs^12,13^.

The color of rumen epithelium tissues on the other hand can be affected by the particle size and chemical composition of the feed, leading to a dark-brownish appearance^14^. This darkening, detected in the keratinized cornified layer, is believed to be a surface phenomenon resulting from the dark color of the rumen fluid due to pelleting total mixed rations^15,16^, or from iron-containing pigments^17^.

Most previous studies have focused on using Moringa leaves as a protein alternative or feed additive. To our knowledge, there is a lack of research on the use of whole Moringa plants as non-traditional forage to replace fiber sources in pelleted total mixed rations and its effects on rumen characteristics. Therefore, this study aims to evaluate the impact of partially or fully replacing alfalfa hay with whole *Moringa oleifera* or *Moringa peregrina* plants as drought-resistant forage in pelleted total mixed rations on growth performance and rumen characteristics in lambs.

## Results

This study evaluates the effects of partial or complete substitution of alfalfa hay with whole Moringa plants *(Moringa oleifera (MO)* and *Moringa peregrina (MP)* incorporated into pelleted complete diets of Hari lambs. Key parameters assessed include growth performance, nutrient digestibility, ruminal fermentation characteristics, and rumen epithelial coloration.

### Growth performance

As detailed in Table 1, the replacement of alfalfa hay with MO or MP had no significant impact (*P >* 0.05) on growth metrics such as average daily gain (ADG), total weight gain, final body weight (BW), or feed conversion ratio (FCR). However, the average daily feed intake (ADFI) was significantly reduced (*P* < 0.05) in lambs receiving diets where alfalfa was partially or fully replaced by MO. These results indicate that whole Moringa plants can replace alfalfa hay in lamb diets without detrimentally affecting growth efficiency or nutrient utilization.

**Table 1:** Assessment of growth performance in Hari lambs fed pelleted diets containing partial or complete replacement of alfalfa hay with *Moringa oleifera* or *Moringa peregrina*.

Variable	Treatments^1^					Dependent effects	
	Control	MO	MP	AO	AP	SEM^2^	*P* -value
Initial BW	22.58	22.58	23.52	21.55	23.51	0.54	0.791
Final BW	44.04	40.97	44.36	41.31	42.64	0.59	0.248
Total gain	21.26	18.39	20.84	19.76	19.13	0.55	0.454
ADG	0.25	0.22	0.25	0.24	0.23	0.01	0.391
ADFI	1.38^ab^	1.48^c^	1.74^a^	1.57^bc^	1.70^ab^	0.03	0.006
FCR	6.79	7.05	7.38	6.74	7.63	0.21	0.615

^a−c^Means within the same row with different superscripts are significantly differ at *P* < 0.05.

^1^Treatment: Control: Alfalfa; MO: *Moringa oleifera*; MP: *Moringa peregrina*; AO: Alfalfa + *Moringa oleifera* and AP: Alfalfa + *Moringa peregrina*.

^2^SEM=Standard error of means for treatments effect.

### Nutrient digestibility

The nutrient digestibility data presents, showing no significant differences (*P >* 0.05) among dietary treatments for dry matter, crude protein, neutral detergent fiber, or acid detergent fibre digestibility (Table 2). Ether extract digestibility, however, was significantly decreased (*P* < 0.05) in groups fed Moringa-based diets. These findings suggest that the inclusion of whole Moringa plants does not adversely affect the overall digestibility of major nutrients, despite reduced ether extract digestibility.

**Table 2:** Evaluation of nutrient digestibility in lambs fed pelleted complete diets with partial or complete replacement of alfalfa hay by whole *Moringa oleifera* or *Moringa peregrina* plants.

Variable	Treatments^1^					Dependent effects	
	Control	MO	MP	AO	AP	SEM^2^	*P*-value
Dry matter	65.09	60.92	68.35	62.25	60.04	1.02	0.11
Crude protein	54.01	56.26	67.01	61.74	60.34	1.84	0.31
Ether extract	82.95^a^	40.83^d^	62.65^bc^	54.08^cd^	75.76^ab^	4.24	0.02
Crude fiber	35.26^b^	24.19^b^	28.69^b^	24.48^b^	55.31^a^	3.69	0.01
Ash	25.56	37.17	30.98	35.26	46.75	3.43	0.28
Organic matter	64.52	66.21	70.92	61.70	63.84	1.27	0.27

^a−c^Means within the same row with different superscripts are significantly differ at *P* < 0.05.

^1^Treatment: Control: Alfalfa; MO: *Moringa oleifera*; MP: *Moringa peregrina*; AO: Alfalfa + *Moringa oleifera* and AP: Alfalfa + *Moringa peregrina*.

^2^SEM=Standard error of means for treatments effect.

### Rumen fermentation characteristics

Postprandial ruminal pH remained stable across all dietary groups (*P >* 0.05), as shown in (Table 3). Analysis of individual volatile fatty acids (VFAs) revealed significant treatment effects (*P* < 0.05): acetic acid concentration was highest in MP-fed lambs (46.39 mmol/L) and lowest in MO-fed lambs (37.38 mmol/L). Propionate levels peaked in the alfalfa group (27.50 mmol/L). Butyric acid was elevated in the MO group (33.91 mmol/L) relative to controls (27.28 mmol/L). Valeric acid concentration was greatest in alfalfa-fed lambs (2.63 mmol/L) and lowest in MO-fed lambs (1.65 mmol/L). Branched-chain VFAs, isobutyrate and isovalerate, were significantly higher in the MP group (2.65 and 3.87 mmol/L, respectively). Despite these variations, total VFA concentrations did not differ significantly (*P >* 0.05) across treatments.

**Table 3:** Rumen fluid pH and fermentation profile in Hari lambs fed diets with *Moringa oleifera* or *Moringa peregrina* as partial or complete substitutes for alfalfa hay.

Variable	Treatments^2^										Dependent effects			
	Con.		MO		MP		AO		AP		SEM^3^			*P*-value
pH														
Pre-pH	6.81		6.53		6.60		6.48		6.38		0.05			0.06
Post-pH	5.68		5.88		5.73		5.80		5.90		0.05			0.64
Volatile Fatty Acids (VFAs)^1^														
Acetic acid	42.05^b^		37.38^c^		46.39^a^		39.28^c^		43.18^b^		0.76			< 0.0001
Propionic acid	25.84^ab^		23.84^b^		18.99^c^		27.50^a^		24.42^b^		0.69			< 0.0001
Butyric acid	27.28^b^		33.91^a^		27.10^b^		28.89^ab^		28.63^ab^		0.76			0.0106
Valerate	2.63^a^		1.65^d^		2.12^bc^		2.24^ab^		1.79^dc^		0.09			< 0.0001
Iso butyric	1.62^b^		0.75^c^		2.65^a^		0.77^c^		0.82^c^		0.18			< 0.0001
Iso valerate	2.23^b^		0.49^c^		3.87^a^		0.55^c^		0.85^c^		0.31			< 0.0001
Total VFAs	25.97		26.19		31.57		30.35		32.12		1.53			0.604

^a−c^ Means within the same row with different superscripts are significantly differ at *P* < 0.05.

^1^Individual VFA given as mol/100mol, and total VFAs as mmol/L.

^2^Treatment: Control: Alfalfa; MO: *Moringa oleifera*; MP: *Moringa peregrina*; AO: Alfalfa + *Moringa oleifera* and AP: Alfalfa + *Moringa peregrina*.

^3^SEM=Standard error of means for treatments effect.

### Rumen epithelium coloration

**Fig. 1:**
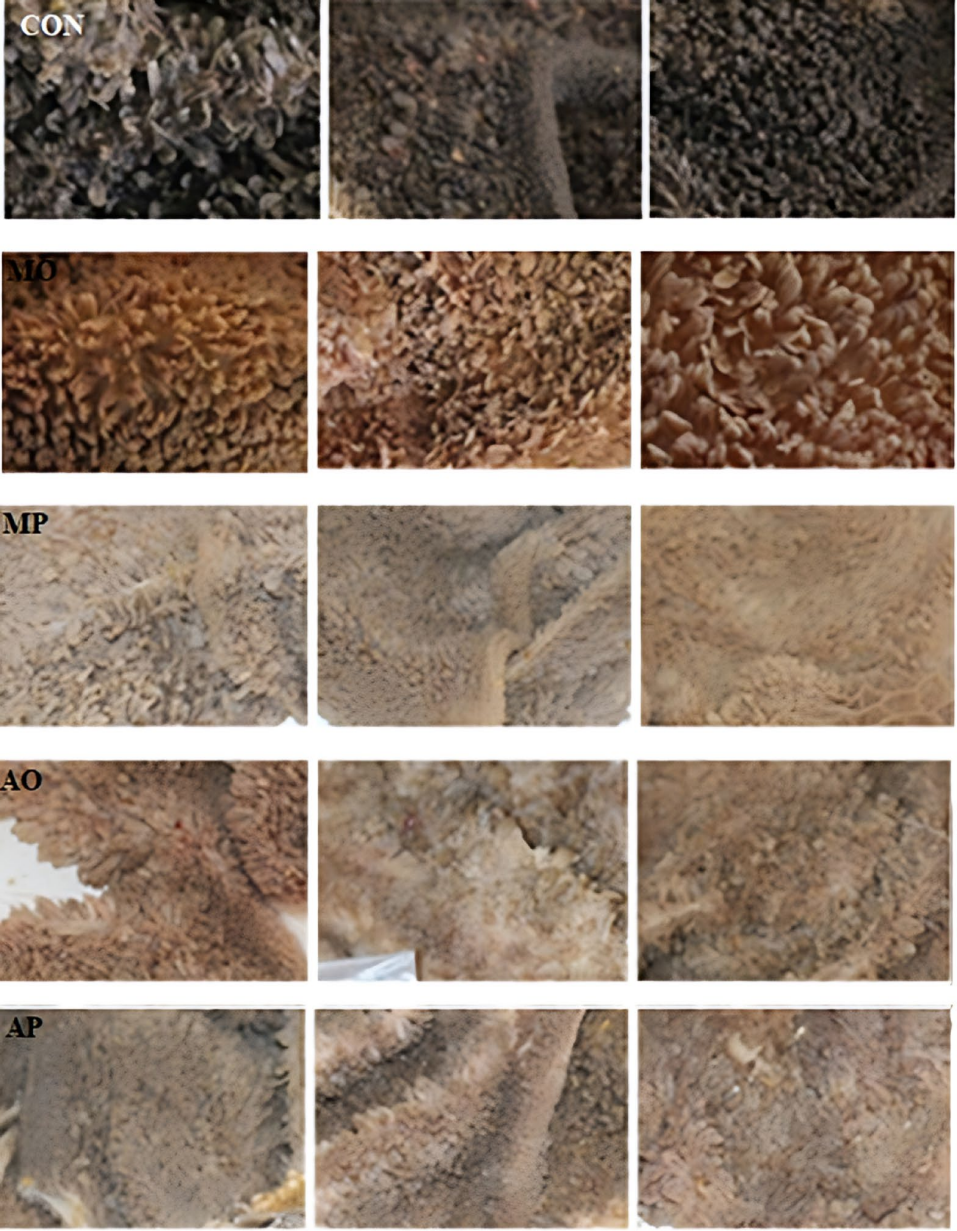
Effects of replacing alfalfa Hay with *Moringa oleifera* or *Moringa peregrina* on rumen epithelium coloration in Hari Lambs. (Con: Alfalfa hay (control); MO: Complete replacement of alfalfa hay with *Moringa oleifera*; MP: Complete replacement of alfalfa hay with *Moringa peregrina*; AO: 50% Alfalfa hay + 50% *Moringa oleifera*; AP: 50% Alfalfa hay + 50% *Moringa peregrina*).

Colorimetric analysis of ruminal epithelial tissue (Table 4) demonstrated significant differences among groups. The lightness parameter (L*) was markedly increased (*P* < 0.05) in lambs fed diets where alfalfa hay was fully replaced by MP (L* = 50.48) or MO (L* = 43.7) compared to the control (L* = 33.62). The red-green chromaticity (a*) remained unchanged among treatments (*P >* 0.05), indicating consistent color intensity. The yellow-blue axis (b*) was elevated in the MP group (15.72), suggesting increased yellow pigmentation relative to the control (9.33), approaching statistical significance (*P* = 0.051). The b/a ratio, indicative of hue balance, differed significantly (*P* < 0.05), with the control exhibiting the highest ratio (20.34), reflecting a predominant yellow hue, while the MO group showed a reduced ratio (6.45), implying a shift towards red coloration. Figure 1 illustrates these differences, with alfalfa-fed lambs exhibiting the darkest rumen epithelium and the MP group the lightest.

**Table 4:** Evaluation of rumen epithelial color changes in Hari lambs fed diets with partial or complete replacement of alfalfa hay by *Moringa oleifera* or *Moringa peregrina*.

Variable^1^	Treatments^2^					Dependent effects	
	Con.	MO	MP	AO	AP	SEM3	*P*-value
L	33.62b	43.7a	50.48a	36.88b	40.57b	1.73	0.002
a	1.56	2.36	2.39	1.61	1.91	0.26	0.123
b	9.33b	13.98ab	15.72a	12.88ab	13.05ab	0.73	0.051
b/a ratio	20.34a	6.45b	7.03b	8.42ab	7.57b	1.71	0.017

^a−c^ Means within the same row with different superscripts are significantly differ at *P* < 0.05.

^1^Color indices: Measured using the Hunter Lab color space on a three-dimensional axes L, a, and b (“L” is light to dark, whereas “a” is green to red, and “b” is yellow to blue) provided numerical descriptor of the color differences between a sample and a standard or target color; b/a ratio is determined by rotation about a and b axes.

^2^Treatment: Control: Alfalfa; MO: *Moringa oleifera*; MP: *Moringa peregrina*; AO: Alfalfa + *Moringa oleifera* and AP: Alfalfa + *Moringa peregrina*.

^3^SEM=Standard error of means for treatments effect.

### Correlation between rumen color and VFAs

**Fig. 2:**
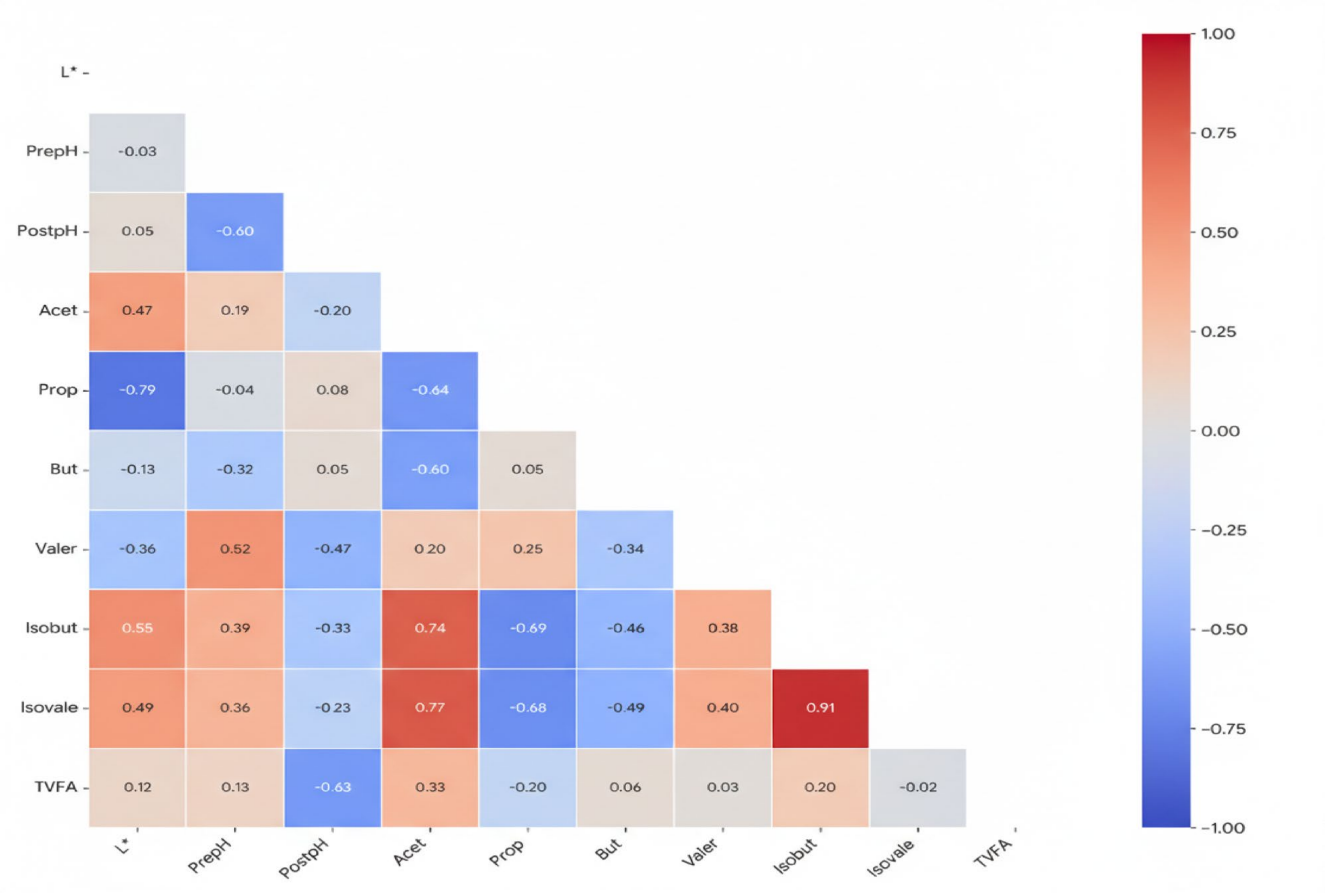
Triangular correlation visualizing the association between ruminal colorimetric values and individual volatile fatty acids in Hari lambs subjected to alfalfa hay replacement with *Moringa oleifera* or *Moringa peregrina* in pelleted diets. L*: Lightness; PrepH: Pre-pH; PostpH: Post- pH; Acet: Acetate; Prop: Propionate; But: Butyrate; Vale: Valerate; Isobut: Isobutyrate; Isovale: Isovalerate; TVFA: Total Volatile Fatty Acids.

The Visualizes the Pearson correlation analysis, revealed significant associations between rumen epithelial lightness (L*) and ruminal VFA concentrations (Fig. 2). Positive correlations were observed between L* and isobutyrate (*r* = 0.551, *P* = 0.012) and isovalerate (*r* = 0.487, *P* = 0.029), indicating that lighter rumen coloration is significantly associated with higher levels of branched-chain VFAs. Conversely, a strong negative correlation was found between L* and propionate concentration (*r* = −0.793, *P* < 0.0001), suggesting that lighter rumen epithelium coloration is associated with lower propionate levels.

## Discussion

Results of the current study indicate that partial or complete substitution of alfalfa hay with whole Moringa plants (*Moringa oleifera and Moringa peregrina*) in pelleted diets for Hari lambs maintained overall growth performance, nutrient digestibility and ruminal pH. The primary changes were observed in the rumen environment, manifesting as significant shift in ruminal volatile fatty acid (VFA) profiles and changes in rumen epithelial coloration.

The absence of significant differences in average daily gain (ADG), total weight gain, final body weight, and feed conversion ratio (FCR) across dietary treatments aligns with previous findings indicating that Moringa spp. can serve as viable forage alternatives in ruminant diets without impairing growth^18^. The significant reduction in average daily feed intake (ADFI) observed in MO-supplemented groups may be attributed to the higher nutrient density or secondary metabolites such as tannins and saponins in Moringa, which can modulate palatability or rumen fermentation^19,20^. However, despite decreased feed intake, growth performance remained unaffected, suggesting efficient nutrient utilization or compensatory feed efficiency in these lambs.

The largely unchanged digestibility of dry matter, crude protein, and fiber fractions corroborates prior reports indicating that Moringa inclusion does not compromise ruminal fermentation or fiber degradation and reduce methane emission^21,22^. The reduction in ether extract digestibility could be related to lipid-protective effects of Moringa’s phytochemicals, which may inhibit ruminal biohydrogenation or alter lipid metabolism^23^. Such effects warrant further investigation to elucidate potential implications for fatty acid profiles in meat and milk.

Stable ruminal pH values across treatments confirm that partial or complete replacement of alfalfa hay with Moringa does not disrupt rumen acid-base balance, which is crucial for microbial homeostasis^24^. The observed variations in individual VFAs specifically elevated acetic acid in MP-fed lambs and increased butyric acid in MO-fed lambs reflect shifts in microbial fermentation pathways influenced by forage type and secondary compounds^25^. The higher branched-chain VFAs (isobutyrate and isovalerate) in MP groups may suggest enhanced protein fermentation or microbial protein synthesis^26^. The lack of significant differences in total VFA concentration implies overall fermentative activity was maintained despite shifts in individual acids.

The significant lightening of rumen epithelial color (higher L* values) in lambs fed Moringa-based diets, especially with MP, suggests morphological or biochemical alterations possibly linked to dietary bioactive compounds^27^. The increased yellow pigmentation (higher b* values) observed in MP-fed lambs could be associated with deposition of carotenoids or flavonoids, known to impart yellow hues and present in Moringa species^28^. The color changes may reflect modulation of rumen epithelial cell turnover or oxidative status, though further histological and biochemical analyses are needed.

Pearson correlations revealed that lighter rumen epithelium was positively associated with branched-chain VFAs (isobutyrate and isovalerate) and negatively correlated with propionate levels. This relationship suggests that shifts in ruminal fermentation toward increased protein degradation (reflected by branched-chain VFAs) might be linked to the observed variation in epithelial pigmentation or tissue composition. Conversely, reduced propionate typically a major gluconeogenic precursor could imply altered energy metabolism in ruminal tissue^29^. Such correlations provide novel insights into the interplay between ruminal fermentation profiles and tissue characteristics, a topic that remains underexplored.

## Conclusions

The present study demonstrates that whole Moringa plants (*Moringa oleifera* and *Moringa peregrina*) can effectively replace alfalfa hay partially or completely in pelleted diets for Hari lambs without compromising growth performance or overall nutrient digestibility, except for a reduction in ether extract digestibility. Although ruminal pH and total volatile fatty acid concentrations remained stable, significant alterations in individual VFA profiles and rumen epithelial coloration were observed, indicating shifts in ruminal fermentation patterns and epithelial tissue characteristics. The positive correlation between rumen epithelial lightness and branched-chain VFAs, alongside the negative association with propionate, highlights the complex interactions between diet, rumen fermentation, and tissue morphology. However, this study has a specific limitation: first, the lack of dietary pigment analysis restricts the ability to rule out confounding factors rumen coloration; and second, the absence of histological examination limits the understanding of precise cellular mechanisms driving these epithelial changes. Therefore, further studies are needed to explore the biochemical and histological mechanisms underlying the observed changes in rumen epithelial coloration, to fully elucidate these tissue responses to dietary Moringa.

## Materials and methods

### Ethical approval and animal welfare

The current study followed animal research guidelines and received approval from the Research Ethics Committee (REC) of King Saud University, Riyadh, Saudi Arabia, under approval number KSU-SE-21-19 > All methods and regulations. The study is reported in accordance with ARRIVE guidelines.

### Study site

The research was conducted at the Experimental Station (Al-Ammariah) of the Animal Production Department, College of Food and Agriculture Sciences, King Saud University, Riyadh, Saudi Arabia (24°48’21.0"N 46°31’14.1"E). Riyadh is known for its hot climate and extreme aridity with minimal rainfall throughout the year.

### Animals and experimental design

Lambs were purchased from a local market and acclimated to the pens and basal diet a 4-week adaptation period at Teaching and Research Farm. The basal diet provided 1.95 Mcal ME and 13.0% CP/kg (on a dry matter basis) and was fed at 2.5% of the animals’ initial body weight, as per NRC (30). The feed was offered twice daily at 08:00 and 15:00 h, and animals had a free access to fresh, clean water. During adaptation, the lambs were vaccinated, treated with prophylactic anthelmintic, and supplemented with multivitamins. The experiment was conducted using 60 male Hari lambs (age: 3–4 months, weight: 22.8 ± 0.36 kg), and lasted for 84 days. The study utilized a completely randomized design (CRD), with animals randomly assigned to five.

groups (four replicates per group, each with three animals). Each group received one of five dietary treatments as in Table 5: T1: alfalfa-based pelleted total mixed diet (TMD), T2: *Moringa oleifera*-based pelleted TMD, T3:*Moringa peregrina*-based pelleted TMD, T4:alfalfa + *Moringa oleifera*-based pelleted TMD, and T5:alfalfa + *Moringa peregrina*-based pelleted TMD. All diets were formulated to meet the nutritional requirements of growing lambs according to NRC^30^.

At the end of the experiment, a metabolic trial was conducted. Four lambs from each treatment group (one per replicate) were placed in metabolism cages for 7 days: 4 days for adaptation and 3 days for sample collection. At the end of the feeding period, lambs were fasted for 16 h and provided with ad libitum water, and final slaughter weights were recorded. Slaughter was conducted at a commercial facility following Halal procedures, involving transection of the jugular veins and carotid arteries without prior stunning. Immediately after evisceration, samples were collected, including 50 mL of rumen liquor and rumen epithelial tissues, for subsequent analysis of ruminal pH, total and individual concentrations of short-chain fatty acid (SCFA), and epithelial coloration.

**Table 5:** Ingredients and chemical composition of the experimental diets (DM basis).

Ingredients (%)	Treatments^1^				
	Control	MO	MP	AO	AP
Alfalfa	40	0	0	20	20
*M. oleifera*	0	40	0	20	0
*M. peregrina*	0	0	40	0	20
Corn	33.1	34.51	34.51	33.65	33.88
Soya bean meal	13	11.59	11.1	12.45	12.3
Wheat bran	12	12	12.49	12	11.92
Premix	0.3	0.3	0.3	0.3	0.3
Salt	0.5	0.5	0.5	0.5	0.5
Limestone	1.1	1.1	1.1	1.1	1.1
Total	100%	100%	100%	100%	100%
Chemical composition:					
DM%	88.51 ± 1.53	88.21 ± 2.01	87.95 ± 1.78	88.92 ± 1.45	89.72 ± 1.53
EE%	2.76 ± 0.16	2.15 ± 0.12	2.52 ± 0.21	2.13 ± 0.11	2.24 ± 0.32
CP%	14.9 ± 0.57	14.53 ± 0.21	15.07 ± 0.10	14.03 ± 0.17	14.45 ± 0.11
ME (Mcal)	3.90 ± 0.30	3.88 ± 0.20	3.98 ± 0.21	3.96 ± 0.36	3.97 ± 0.45

^1^Treatment: Control: Alfalfa; MO: *Moringa oleifera*; MP: *Moringa peregrina*; AO: Alfalfa + *Moringa oleifera* and AP: Alfalfa + *Moringa peregrina*.

^2^ DM: Dry matter, EE: Ether extract, CP: Crude protein, ME: Metabolizable energy.

Premix: is a commercial vitamin-mineral mix containing per kg: 10,000 IU vit A, 1000 IU vit D, and 20 IU vit E, as well as 300 mg Mg, 24 mg Cu, 0.6 mg Co, 1.2 mg I, 60 mg Mn, 0.3 mg Se, and 60 mg Zn.

### Experimental measurements

#### Feed analysis

Feed samples were collected weekly and stored at −20 °C to prevent spoilage. After the study, the samples from each treatment were homogenized and subjected to proximate nutrients analysis. Parameters such as dry matter (DM), ash, crude protein (CP), neutral detergent fibre (NDF), and acid detergent fibre (ADF) were determined using AOAC^31^ Official methods. CP was measured using the Kjeldahl procedure using AOAC^31^guidelines, and NDF and ADF were measured as described by Van Soest et al.^33^.

#### Performance data

To assess dry matter intake (DMI), average daily gain (ADG), and feed efficiency (FE), feed offered and refusals were weighed weekly, while live body weight was measured every two weeks. Daily feed intake was calculated on a DM basis. On day 1, and then every 2 weeks until the end of the study, lambs were weighed before morning feeding at 07:30 h using an electronic scale. ADG was calculated as the difference between final and initial body weights divided by the number of experimental days. The gain-to-feed ratio was calculated and expressed as body weight gain per kg of DMI to evaluate FE.

#### Nutrients digestibility

During the metabolic trial, daily feed offered and refusals were recorded to determine feed intake, and daily excreted feces weights were recorded to determine the amount of nutrients voided in the feces. Over 3-days sample collection period, 5% of feed refusals and 20% of feces amounts were sampled, and stored at −20 °C. The samples collected from one animal were then pooled and analyzed for DM, crude fiber (CF), crude protein (CP), ether extract (EE), and ash content using AOAC standard methods. Samples were thawed, air-dried at 60 °C, and ground to pass through a 1 mm sieve. DM content was determined by drying samples at 105 °C for 24 h, while ash content was assessed by combusting samples in a muffled furnace at 550–600 °C for 6 h. CF was determined by treating samples with boiling acid and alkali solutions. CP content was measured using the Kjeldahl method according to AOAC^32^ guidelines.

#### Rumen liquor and rumen epithelium coloration

The collected rumen liquor samples were filtered through four layers of cheesecloth. Immediately, pH of the samples was measured using digital pH meter. Subsequently for VFAs analysis, 8 mL of the filtered rumen liquor was transferred to acid-resistant plastic test tubes containing 2 mL of 1 N sulfuric acid and stored at −20°C for analysis of volatile fatty acid (VFA) concentrations via using gas chromatography with a Nukol column (Supelco™ WSAF-2 Mix from SUPELCO Co., Bellefonte, PA, USA), following the procedure by Erwin et al.^34^.

The color of the rumen epithelium and pigmentation, tissues samples (approximately 5 * 5 cm) were excised from the ventral sac of rumen immediately after slaughter. These samples were rinsed with physiological saline to remove feed particles and blotted dry. Color coordinates (L*, a*, b*) were then assessed using a Minolta Chroma Meter (Konica Minolta CR-400, Japan). Prior to use, the device was calibrated against a standard white tile(Y = 93.5, x = 0.3132, y = 0.3198). mesurements were taken by using the stanserd illuminant D65 with a 2° standard observer angle, following the CIELAB^35^ system (L* for lightness, and a* and b* for color coordinates).

#### Statistical analysis

The current experiment was conducted using completely randomized design (CRD). All data were analyzed using one-way ANOVA with the general liner model (GLM) procedure in SPSS software version 22 (SPSS Inc., Chicago, IL, USA). The statistical model used was:

$$Y_{{ij}} = {\text{ }}\mu {\text{ }} + {\text{ }}T_{i} + {\text{ }}e_{{ij}} ,$$

where ***Y***_***ij***_ is the observationof the ***j*** is the lamb, ***I*** is the treatments, ***µ*** *is the overall mean*, ***T***_***i***_ *is the fixed effect of dietary treatment (i = 1 to 5)*,* and* ***e***_***ij***,_ *is the random error associated with observation.* To determine significant differences between means of groups, Tukey’s test was applied, with significance set at *p* ≤ 0.05. Additionally, the linear relationship between selected variables were assessed using Pearson correlation test.

## Data Availability

The data presented in this study are available upon request from the corresponding author.
